# Patient and public involvement in abortion research: reflections from the Shaping Abortion for Change (SACHA) Study

**DOI:** 10.1136/bmjsrh-2023-202018

**Published:** 2024-02-09

**Authors:** Rebecca Blaylock, Maria Lewandowska, Charlotte Kelly, Becky Gunn, Rebecca Meiksin, Rachel H Scott, Melissa J Palmer, Kaye Wellings, Patricia A Lohr, Rebecca S French, The SACHA Study Team N/A

**Affiliations:** 1 Centre for Reproductive Research & Communication, British Pregnancy Advisory Service, London, UK; 2 Department of Public Health, Environments and Society, London School of Hygiene and Tropical Medicine, London, UK; 3 PPI representatives, London, UK; 4 Department of Population Health, London School of Hygiene and Tropical Medicine, Faculty of Epidemiology and Population Health, London, UK; 5 London School of Hygiene & Tropical Medicine, London, UK

**Keywords:** abortion, induced, Patient Participation, research design, Sexual Health, Reproductive Health

## Abstract

Patient and public involvement (PPI) is limited within abortion-related research. Possible reasons for this include concerns about engaging with a stigmatised patient group who value confidentiality and may be reluctant to re-engage with services. Structural barriers, including limited funding for abortion-related research, also prevent researchers from creating meaningful PPI opportunities. Here, we describe lessons learnt on undertaking PPI as part of the Shaping Abortion for Change (SACHA) Study, which sought to create an evidence base to guide new directions in abortion care in Britain.

Two approaches to PPI were used: involving patients and the public in the oversight of the research and its dissemination as lay advisors, and group meetings to obtain patients’ views on interpretation of findings and recommendations. All participants observed the SACHA findings aligned with their own experiences of having an abortion in Britain. These priorities aligned closely with those identified in a separate expert stakeholder consultation undertaken as part of the SACHA Study. One additional priority which had not been identified during the research was identified by the PPI participants.

We found abortion patients to be highly motivated to engage in the group meetings, and participation in them actively contributed to the destigmatisation of abortion by giving them a space to share their experiences. This may alleviate any ethical concerns about conducting research and PPI on abortion, including the assumption that revisiting an abortion experience will cause distress. We hope that our reflections are useful to others considering PPI in abortion-related research and service improvement.

**Key points**

- Abortion patients were highly motivated to engage in patient and public involvement (PPI) activities, and participation in them actively contributed to the destigmatisation of abortion.
- We advise others to be mindful of the risks of using social media for abortion-related research and PPI.
- PPI in abortion research has been lacking, but this process shows that it is feasible and mutually beneficial to PPI participants and researchers.

## Background

Patient and public involvement (PPI) – activities where members of the public or patients are actively involved in contributing their perspectives as advisers or co-researchers – is limited within abortion-related research. Possible reasons for this include concerns from researchers about engaging with a stigmatised patient group who value confidentiality and may be reluctant to re-engage with services.^1 2^ Structural barriers, including limited funding for abortion-related research, also prevent researchers from creating meaningful PPI opportunities.^3^ Here, we describe our reflections on undertaking PPI as part of the Shaping Abortion for Change (SACHA) Study.

The aim of the SACHA Study was to provide an evidence-base to guide new directions for abortion care in Britain. Two approaches to PPI were used in the study: involving patients and the public in the oversight of the research and its dissemination as lay advisors, and group meetings to obtain patients’ views on interpretation of findings and recommendations.^4^ We have used the GRIPP2 checklist to guide the reporting of these PPI activities.^5^

### Why and how did we involve patients and the public?

At the start of the study, two PPI representatives (CK and BG) were recruited via the Royal College of Obstetricians and Gynaecologists’ PPI network, ‘Women’s Voices’. These representatives, who had an interest in abortion but no medical background or involvement in service provision, were consulted at all stages of the project and were members of the study advisory group. They helped shape patient information materials and data collection tools and worked with the research team to identify key findings and present them in an accessible way. They were reimbursed in accordance with National Institute for Health and Care Research (NIHR) guidance.^6^

Together with the Centre for Reproductive Research & Communication (CRRC) at the British Pregnancy Advisory Service (BPAS), the SACHA team also undertook virtual group meetings with a small number of patients who had recently had an abortion. These meetings allowed the research team to share findings from the SACHA Study with abortion patients, seek feedback on whether the results resonated with their experiences, and get their views on which of the study’s recommendations they would prioritise for future research and service development.

### What did we do?

The CRRC Research and Engagement Lead (RB) recruited participants for the group meetings from a pool of BPAS patients who had their abortions in April and May 2022. Details of the opportunity were also circulated via a Scottish abortion advocacy group on social media. Those interested in participating were directed to an online recruitment tool which asked them about their sociodemographic characteristics, the method of their most recent abortion, contact details, and availability. RB contacted potential participants and invited them to the group meeting, ensuring representation of a range of ages, ethnicities, and experiences of abortion methods.

ML and RF hosted three 1-hour group meetings over Zoom. Meetings were focused on key themes identified in the SACHA Study findings by the research team and PPI representatives in the study advisory group (CK and BG). These were: (1) patient-centred care, (2) roles of health professionals and (3) law and regulation. ML prepared lay summaries for each theme and shared them prior to the meeting to facilitate a discussion based on the following questions:

- Why were you interested in taking part in today’s discussion?
- Was there anything in the findings that immediately struck you?
- What rang true from our findings? What’s missing?
- What do you think is the most important thing we should be recommending to policymakers, service providers and other researchers?

Participants were given the opportunity to use a pseudonym, could choose to have their camera on or off, and were reminded of the importance of respecting each other’s confidentiality. It was made clear to those participating that they did not have to share any personal details or experiences during the meeting. The discussions were not audio-recorded, but detailed notes were taken, and reflections documented at the end of each group. All participants were thanked for their time with a £20 shopping voucher. Meetings were held in the afternoon during office/school hours and the evening to ensure those with different commitments could attend.

Ten patients participated in the three group meetings. Their sociodemographic characteristics are described in table 1.

**Table 1:** Sociodemographic characteristics and most recent abortion method of patient and public involvement (PPI) participants.

Characteristic	n (N=10)
Ethnicity	
White (any)	7
Black (any)	2
Mixed (any)	1
Age (years)	
18–24	3
25–34	5
35–44	2
Most recent abortion method	
Medical abortion	7
Surgical abortion	3

### What did we learn about patient perspectives on the SACHA findings?

All participants observed that the SACHA findings aligned with their own experiences of having an abortion in Britain.^7^ Priorities that the PPI group identified for future research and service development were:

- Improving access to abortion through all healthcare settings, especially within sexual and reproductive health services.
- Better signposting to specialist abortion services by primary care, pharmacies, and other healthcare settings, and in online spaces.
- Nurses and midwives providing abortions.
- Incorporating abortion into school Sex and Relationship Education (SRE) curricula.
- Improving support for abortion patients in education and work settings, including paid leave.
- More opportunities for peer support throughout and after abortion treatment.
- More information to manage patient expectations around bleeding and pain during the abortion, and provision of information for others who may be providing support (eg, partner, friends or family).
- Better post-abortion follow-up by providers.

These priorities aligned closely with those identified in a separate expert stakeholder consultation undertaken as part of the SACHA Study. The only additional priority which had not been identified during the research was a need to address a lack of support and information in educational and work settings – an important recommendation that would not have been identified without this exercise.

### Our reflections on opportunities and challenges of PPI in abortion research

Contrary to our initial concerns about the reluctance of abortion patients to be involved in PPI activities, we found them to be highly motivated to engage. Participants described their desire to help others having abortions in the future as a key motivation for involvement. Although they were told that we were only seeking their perspectives on our findings, all were keen to share their personal experiences. We identified a therapeutic quality to the group meetings which created a space for participants to share their experiences which some had previously not done, or if they had, were met with stigmatising responses. In this sense, the group meetings appeared to actively contribute to the destigmatisation of abortion among the participants.^8^

McDonagh *et al*, among others, have highlighted the importance of including individuals from minoritised groups in PPI efforts while acknowledging that they are offering their own perspectives and should “not be expected to be *representative”* of those groups.^4^ Roughly one-third of our participants were from racialised and minoritised ethnic groups. We used the CRRC recruitment system whereby all patients who have an abortion at BPAS are asked whether they consent to be contacted about future research. Approximately 36 000 patients agree per year, representing 35% of the total patient population. This has improved the diversity of research populations at the organisation in recent years and is a strength of the research function at BPAS which may not be available to all. However, researchers must consider how to include seldom-heard voices in PPI activities.

We faced some challenges through advertising the PPI opportunity via social media, as our recruitment tool was sabotaged with responses from suspected anti-choice activists and ‘bots’. This made ascertaining genuine respondents difficult. Therefore, the decision was made to limit participation to BPAS patients to ensure a safe space for discussion. We advise others who are recruiting for both research and PPI to be mindful of the risks of using social media for abortion-related work, and consider mitigation strategies to avoid exposing themselves or their participants to anti-choice activity.^9^

## Conclusions

Involving patients and the public throughout the research process enabled the SACHA Study research team to design patient-centred data collection tools, seek feedback on the study findings, and set patient-centred priorities for future research and service development. We found abortion patients to be highly motivated to engage in the group meetings, and participation in them actively contributed to the destigmatisation of abortion by giving them a space to share their experiences. This may alleviate any ethical concerns about conducting research and PPI on abortion including the assumption that revisiting an abortion experience will cause distress. We hope that our reflections are useful to others considering PPI in future abortion-related research and service improvement.
